# Climate change shapes the future evolution of plant metabolism

**DOI:** 10.1002/ggn2.10022

**Published:** 2020-08-10

**Authors:** Sophia Y. Xu, Jing‐Ke Weng

**Affiliations:** ^1^ Whitehead Institute for Biomedical Research Cambridge Massachusetts USA; ^2^ Department of Biology Massachusetts Institute of Technology Cambridge Massachusetts USA

**Keywords:** climate change, growth‐defense balance, metabolic engineering, plant metabolism, specialized metabolism, synthetic biology

## Abstract

Planet Earth has experienced many dramatic atmospheric and climatic changes throughout its 4.5‐billion‐year history that have profoundly impacted the evolution of life as we know it. Photosynthetic organisms, and specifically plants, have played a paramount role in shaping the Earth's atmosphere through oxygen production and carbon sequestration. In turn, the diversity of plants has been shaped by historical atmospheric and climatic changes: plants rose to this challenge by evolving new developmental and metabolic traits. These adaptive traits help plants to thrive in diverse growth conditions, while benefiting humanity through the production of food, raw materials, and medicines. However, the current rapid rate of climate change caused by human activities presents unprecedented new challenges to the future of plants. Here, we discuss the potential effects of modern climate change on plants, with specific attention to plant specialized metabolism. We explore potential avenues of future scientific investigations, powered by cutting‐edge methods such as synthetic biology and genome engineering, to better understand and mitigate the consequences of rapid climate change on plant fitness and plant usage by humans.

## Graphical abstract

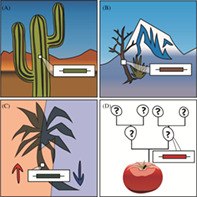

## 1 INTRODUCTION

Plants make up an estimated 80% of Earth's biomass,^1^ and we humans rely on plants for our basic existence. From generating the air we breathe and the food we eat to supplying the raw material for the roofs over our heads and the clothes on our backs, plants and plant products are inextricable from everyday human life. Beyond these basics, the plant kingdom produces an incredible suite of specialized metabolites, also called secondary metabolites, which they use for internal and external signaling, attracting pollinators and seed dispersers, and defending against herbivores and pathogens, among a multitude of other activities.^2^, ^3^, ^4^

**FIGURE 1:**
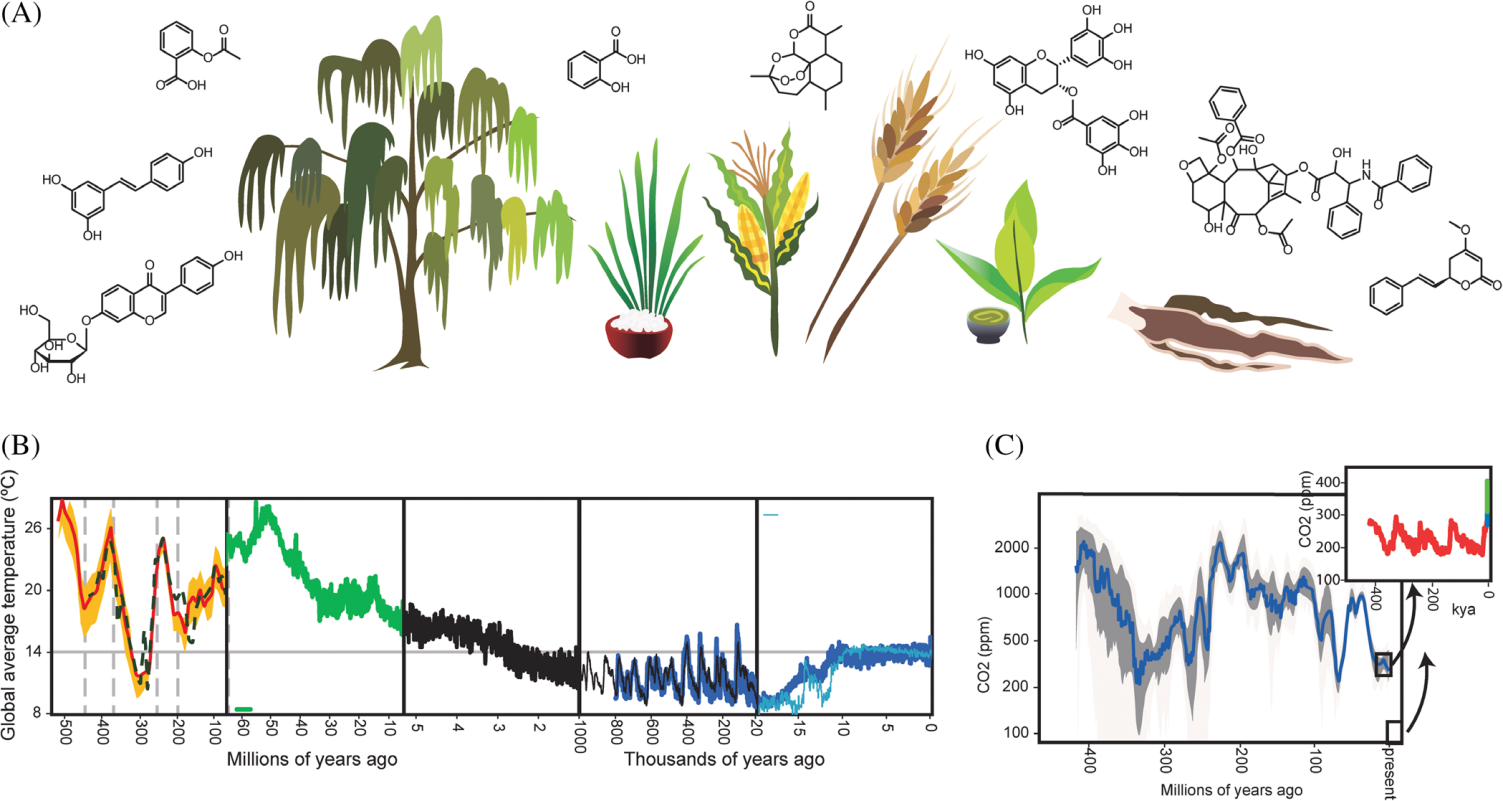
Historical and current climate change and its potential impact on plant metabolism. A, Climate change affects the output of commercially and culturally valuable plants and plant metabolites, some of which are depicted here. These metabolites are used by plants for their own immunity and growth‐defense balance, and are used by humans in food, medicine, and cosmetics, among other fields. B, Global average temperatures extrapolated from the geologic record show dramatic fluctuations in the 500 million years since the estimated rise of land plants. Vertical gray dashed lines indicate major extinction events. Plot is modified from Reference 147 with data from References 148, 149, 150, 151, 152. C, Estimates of global CO_2_ levels in the last 500 million years since the rise of land plants show dramatic fluctuations. Plot is adapted from Reference 153. CO_2_ inset shows measurements compiled from ice cores for the last 500 thousand years as well as more recent measurements taken at the Mauna Loa observatory. Plot was generated with data from References 154, 155, 156, 157, 158, 159

Many of these specialized metabolites have been explored and subsequently repurposed by humans for our own uses. From personal grooming to medicine, the list is extensive (Figure 1A). For example, we use a plethora of characteristic flavor and fragrance compounds extracted from plants to enhance the taste and odor of a variety of dietary and consumer products.^5^, ^6^, ^7^, ^8^ Several classes of colored compounds, including flavonoids and betalains, are used as natural dyes.^9^, ^10^, ^11^, ^12^ Moreover, resveratrol from red wine, anthocyanins in berries, and catechins from tea (*Camellia sinensis*) exhibit a multitude of bioactivities with potential benefits for human health.^13^, ^14^, ^15^, ^16^ Most traditional medicines and a handful of modern medicines are also plant‐derived.^17^, ^18^, ^19^, ^20^ For example, willow (*Salix* spp.) bark is traditionally chewed to alleviate general pain, which led to the development of aspirin, an analog of the willow‐derived compound salicin.^21^, ^22^ Another prominent case is artemisinin, a potent antimalarial which was isolated from sweet wormwood, *Artemisia annua*, a Chinese medicinal plant traditionally prescribed to treat symptoms of malaria.^23^ Yet another example of modern medicines derived from plant precursors is diosgenin, which is extracted from the Mexican yam, *Dioscorea mexicana*, at large scale and subsequently used to manufacture most modern steroidal drugs, including hormonal contraceptives and corticosteroid anti‐inflammatory agents.^24^

Human life is highly dependent upon the rich bio‐ and chemo‐diversity of plants in ways far surpassing the brief list above. However, the anthropogenic acceleration of climate change in the modern era presents a mounting threat to Earth's flora. Forest fires, which are increasing globally, not only destroy the very plants that produce oxygen, but also release a significant amount of sequestered carbon back into the atmosphere, fueling downstream environmental changes.^25^ Warmer temperatures melt permafrost, leading to the appearance of massive sinkholes in the Arctic, which in turn results in the release of large amounts of sequestered methane, a more potent greenhouse gas than carbon dioxide (CO_2_), back into the atmosphere.^26^ Atmospheric concentrations of CO_2_, which have remained relatively stable for the last 800 000 years in the range of 200‐300 ppm, have been creeping up since the first industrial revolution, recently passing a historic high of 400 ppm in 2015 and continuing to climb.^27^, ^28^, ^29^, ^30^ The average annual increase, which in recent years has surpassed 2 ppm/y, may seem insignificant, but is up to two orders of magnitude faster than is recorded in the geologic record (Figure 1B,C).^28^, ^31^ Damage to the stratospheric ozone layer has also led to elevated intensity of ultraviolet (UV) radiation and ozone levels on the ground.^32^, ^33^, ^34^

Many plants, including some producers of valuable specialized metabolites, are sensitive to their growing conditions and are therefore threatened by rapid climate change. For example, important commercial crops such as coffee (*Coffea arabica* and *C canephora*), tea, wine grape (*Vitis* spp.), sugar cane (*Saccharum* spp.), rubber trees (*Hevea brasiliensis*) and oil palms (*Eleis* spp.) have undergone artificial selection by humans to favor certain growth traits at the expense of defense and stress responses.^35^, ^36^, ^37^ Other plants that are the sources of various commercial materials, such as mangroves (*Rhizophora* spp.), have adapted to niche environments, and therefore face habitat loss.^38^ Climate change can influence plant growth, thus affecting the quantity and quality of the specialized metabolites produced. For example, flowering time has been shown to be extremely responsive to environmental stress, potentially via a signaling pathway mediated by simple sugars such as sucrose and glucose.^39^, ^40^, ^41^, ^42^, ^43^, ^44^, ^45^, ^46^ Additionally, the flavor of tea can vary widely depending on growth conditions.^47^, ^48^ Similarly, the richness of the flavor profile of coffee correlates strongly with altitude, but the ideal altitude ranges have been shown to be changing and becoming more inconvenient for farmers in recent years.^49^, ^50^, ^51^, ^52^, ^53^, ^54^ Kava, *Piper methysticum*, a medicinal plant native to the Polynesian islands, produces a bouquet of bioactive kavalactones, and its root is used for preparing a beverage with relaxing effects.^55^ The growth of kava plants is highly sensitive to soil composition, temperature and humidity. The rapidly changing climate therefore raises concerns about the continued viability of kava among other important crops which have served as primary sources of valuable specialized metabolites.

In the estimated 470 million years of land plant evolution,^56^ plants have tolerated and overcome more extreme challenges than present‐day temperatures and CO_2_ levels (Figure 1B,C). For example, atmospheric CO_2_ levels at the time when plants first transitioned from water to land were about 4000 ppm, an order of magnitude higher than today (Figure 1C).^57^, ^58^, ^59^ Notably, plants, as carbon‐fixing organisms, can grow more robustly in the presence of higher CO_2_ levels.^60^ However, many plants may fail to compensate for the accompanying changes such as elevated temperature, humidity, ground ozone, and UV radiation.^28^ As seen in the geologic record, dramatic climate changes such as these often precede mass extinctions (Figure 1C).^61^ Any potential catastrophic loss of floral diversity and accompanying metabolic traits could in turn lead to an existential threat for humanity. However, while the rapid advance of human industry in the past two centuries has contributed in large part to the current looming climate crisis, it has also yielded a wealth of scientific and technological advances that could be harnessed to protect the combined future of plant and human life.

Below, we provide our perspectives on the ways in which climate change directly and indirectly affects plant metabolism, productivity, and other relevant growth traits, and how we could use modern technologies to ameliorate these effects.

## 2 ENVIRONMENTAL FACTORS INFLUENCE PLANT SPECIALIZED METABOLISM

Many specialized metabolic processes in plants are regulated by environmental factors, as demonstrated by an extensive body of research in the past decades.^62^ For example, light induces a subset of phenylpropanoid production in plants, including flavonoids and hydroxycinnamoyl glycosides known to be involved in UV protection and defense.^63^, ^64^ Meanwhile, in response to heightened light intensity, the indole alkaloid camptothecin increases in leaves and decreases in roots in *Camptotheca acuminata*.^65^ Temperature also influences some specialized metabolites: some phenylpropanoids and flavonoids are more abundant at lower growth temperatures,^66^, ^67^, ^68^ while some other compounds, such as the alkaloids conferring bitter flavor in carrots (*Daucus carota*), accumulate at higher temperature.^69^ Other climate‐change‐related parameters, such as elevated ozone and UV radiation, lead to enhanced production of flavonoids, including rutin and quercetin, in soybean (*Glycine max*) plants,^70^ but suppresses terpene production in peaches (*Prunus persica*) and Asterids, among others.^71^, ^72^, ^73^ For terpenes, in addition to environment‐induced changes in terpene biosynthesis, accumulation, and related gene expression, elevated ground‐level ozone is hypothesized to also directly react with many terpenes to generate a host of gaseous and particulate oxygenated compounds, which negatively affect both plant fitness and human health.^74^

While the above studies inform the impact of individual environmental stressors on specific metabolic pathways, they lack the context of complex real‐world climate changes that involve simultaneous variations of multiple environmental factors over dynamic time scales. To this end, Mikkelsen et al recently investigated the effect of varying multiple climate‐change‐related environmental parameters on the accumulation of specialized metabolites in barley (*Hordeum vulgare*), and its relationship with plant pathogen resistance.^75^ They found that elevated CO_2_, ozone, and temperature each increased the resistance of barley to powdery mildew (*Blumeria graminis* f. sp. *hordei*.) infection, but collectively negated any additional resistance. The authors further examined metabolites related to cell wall maintenance, since the route of infection of powdery mildew is through the cell wall. They noticed that changing environmental factors delayed the production of defense metabolites to reinforce the cell wall. Future studies of how various plant metabolic systems respond to complex multifactorial environmental changes, as well as a deeper mechanistic understanding of the integrated genetic circuits underlying these responses, are urgently needed and will enlighten future efforts to engineer desirable plant metabolic traits for the rapidly changing climate.

## 3 THE PLANT GROWTH‐DEFENSE BALANCE COULD BE UPSET BY RAPID CLIMATE CHANGE

Plants grow less when under biotic or abiotic stresses. This tradeoff response is intuitive: plants have access to a finite pool of resources and must decide whether to allocate those resources toward growth or defense. This “growth‐defense balance” evolved over millions of years as an essential survival mechanism in wild plants. However, in human‐cultivated varieties, this trait is likely to have experienced intense artificial selection to maximize biomass production under relatively stable growing environments. The current pace of anthropogenic climate change is faster than has been seen before and may exceed the rate at which evolution can compensate. As such, these new changes may profoundly cripple this fine balance for many wild and crop plants, resulting in significant negative consequences for yields.

Examples of the growth‐defense balance can easily be seen in the field and have been quantified using seed and biomass production and various other measures. For example, drought stress, either alone or in concert with temperature or nutrient stress, decreases seed yield in soybean, pea, and other plants.^76^, ^77^, ^78^ In terms of temperature, an increasing average global temperature is accompanied by a coincident increase in extreme temperature swings.^79^ High temperatures stunt plant growth and pose a dual challenge to plant immunity: not only does the plant's own ability to generate an immune response decrease, but also the virulence of pathogens, represented in one case study by the bacterium *Pseudomonas syringae*, can increase.^80^ In addition, high temperatures in wet areas lead to high humidity, which has been shown to compromise plant immune responses to bacterial pathogens.^81^ Other biotic and abiotic stresses, including pathogen infection, herbivory, drought and UV irradiation, have been shown to adversely affect crop productivity in various plants.^70^, ^82^, ^83^, ^84^ In contrast, the rising atmospheric CO_2_ level alone was shown to not only promote growth, crop productivity and plant water‐use efficiency,^85^, ^86^ but also prime plant defense against biotic stresses,^87^ illustrating one positive outcome of increasing global CO_2_ levels for plant growth.

However, a recent modeling study predicts that these positive influences on plant growth would be compromised by other aspects of climate change, subsequently affecting overall productivity and imposing carbon penalties on nutrient content.^88^, ^89^

The growth‐defense balance is mediated by complex coordinated actions of several phytohormones and their downstream signaling pathways.^90^, ^91^ For instance, elevated atmospheric CO_2_ levels induce plant stomatal closure through abscisic acid (ABA) signaling, contributing to enhanced water‐use efficiency.^92^ On the other hand, CO_2_‐induced defense priming is partly orchestrated by the plant defense hormone salicylic acid (SA), and is additionally linked to the redox signaling pathway.^87^ A current effort in the field focuses on identifying key regulators that facilitate decoupling of beneficial plant stress responses and their associated negative impacts on plant productivity. For example, the plant defense hormone jasmonate (JA) plays an important role in regulating growth‐defense balance by promoting the production of diverse defense compounds and simultaneously inhibiting growth. This is achieved through the JASMONATE ZIM‐DOMAIN (JAZ)‐MYC transcriptional module.^93^ By generating quintuple and decuple mutants of the 13 JAZ proteins in *Arabidopsis*, Guo et al recently showed that JAZ family members promote biomass accumulation by repressing constitutive immune responses.^94^ Most interestingly, although higher‐order mutants accumulated less biomass at maturity, a quintuple mutant grew at the same relative growth rate as wild‐type plants while exhibiting enhanced defense against insects. Developing mechanistic understandings of the growth‐defense balance under various environmental assaults will be crucial for improving the growth robustness of plants against more variable environments.

## 4 HARNESSING WORLD PLANT DIVERSITY TO BUILD MORE RESILIENT CROP PLANTS

**FIGURE 2:**
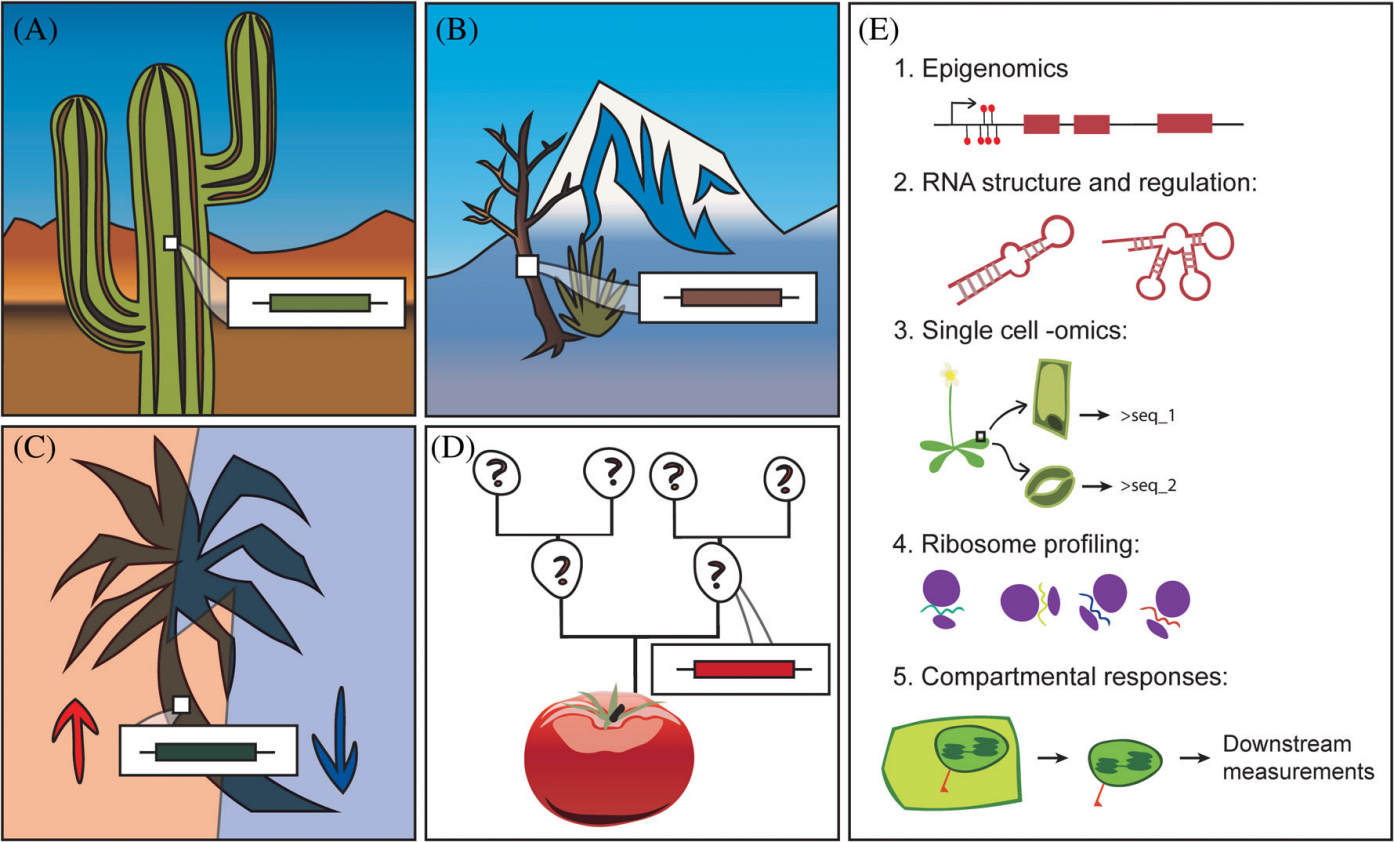
Several approaches to understand and engineer stress tolerance in plants amid climate change. Understanding the ways in which extant plants can respond to abiotic and biotic stresses can guide future work to develop plants which are more resilient to rapidly changing growing conditions. Populations of plants which are potentially interesting to focus on include those that grow in extreme environments (A, B), single species that show differential gene expression in different environments (C), and wild varieties of commercial cultivars (D). (E) The application of modern sequencing and other novel omics technologies to study epigenomes, RNA structures, single cells, ribosomes, and subcellular compartments may provide deeper insights into mechanisms of stress tolerance in diverse plants

Despite ongoing warnings about significant changes in climatic parameters, plants have persisted through more dramatic changes on Earth over the past hundreds of millions of years and will continue to adapt to new environmental conditions at their own pace. However, human reliance on plants and plant products drives us to seek solutions to maintain plant biodiversity and productivity in light of the changing climate. We may consider searching for solutions among plant extremophiles. Take, for example, desert plants, which grow under extreme temperatures, higher light intensity, and low water conditions, or plants which grow at high altitudes where the air is thinner and oxygen is less abundant (Figure 2A,B).^95^, ^96^, ^97^ Further temperature tolerance mechanisms may be gleaned from Antarctic plants, or plants that have survived dramatic events such as forest fires and prolonged flood.^98^, ^99^, ^100^ Some plant extremophiles have evolved to accumulate certain specialized metabolites at very high levels as part of their unique adaptive strategies, with a few already harnessed for human uses, including UV protection, food, and beverage.^101^, ^102^, ^103^ The rich genetic and biochemical bases underlying each case of these amazing adaptations await discovery.

By investigating various mechanisms of how plant extremophiles respond to and defend against various environmental stressors, we may use modern genetic engineering techniques to transplant some of the mechanisms into other plants to gain desirable resistance traits.^104^, ^105^ Temperature sensitivity may be a good place to start, illustrated by the following three examples. First, recent research has revealed a new temperature‐sensing mechanism in plants: the rate of conversion of the phytochrome photoreceptors between the red and far‐red light‐absorbing forms.^106^, ^107^, ^108^ Therefore, plant temperature resistance can potentially be augmented by developing crops with variable phytochrome conversion rates, although more careful investigation of the interplay between light and temperature sensing must be conducted. Second, in plants that evolved to withstand the extreme cold of winter, various ice‐binding proteins (IBPs) have been discovered which suppress the formation of ice crystals in cells.^109^, ^110^ Third, in *Selaginella lepidophylla*, colloquially called the resurrection plant and one of the only few Selaginellaceae species that evolved the ability to revive from an entirely dried state, accumulation of high levels of drought‐resistant sugars, such as trehalose, as well as several other antioxidant metabolites, has been attributed to the plant's ability to “resurrect” from complete desiccation.^111^, ^112^ Plant IBPs and specific anti‐drought metabolite pathways from plant extremophiles thus present tools for engineering plant resilience under harsh environments.

To elucidate unknown adaptive mechanisms underpinning plant environmental tolerance, particularly in nonmodel plants, we may consider comparing closely related species or ecotypes of the same species that have adapted to different environments (Figure 2C). For example, a recent study compared seedling growth of three related cactus species under differential light and humidity conditions and found significantly different growth performance across them.^113^ Similarly, various ecotypes of the biomass crop *Arundo donax*, commonly known as the giant reed, exhibit different productivities under drought stress.^114^ Stemming from these initial findings, it may be possible to identify stress‐tolerance‐related candidate genes and pathways by correlating differential gene expression with variable phenotypic performances under specific stress conditions across a diversity panel. Moreover, more effort could also be directed to research in those so‐called “wild” relatives of commercial crops (Figure 2D). Domesticated plants have undergone artificial selection to favor traits that promote growth or prolong shelf life, often at the expense of stress resistance and other “soft” traits, such as flavor.^115^ By studying wild cousins of modern crop plants, we could identify those lost resilience traits and reintroduce them back into crops. Some of these traits indeed involve production of specialized metabolites playing roles in biotic or abiotic defenses, which in turn may improve or diversify those “soft” qualities in derived plant products.^116^

## 5 EPIGENETIC MECHANISMS CONTRIBUTE TO PLANT ADAPTATION UNDER CLIMATE CHANGE

In addition to plant adaptation through genetic changes, epigenetic mechanisms may also play an important role in plant phenotypic variation under rapid climate change (Figure 2E). Epigenetic chromatin and DNA modifications have been shown to influence plant metabolism and stress tolerance.^117^, ^118^, ^119^, ^120^, ^121^ For instance, in clonally propagated white clover *Trifolium repens*,^122^ apomictic dandelion *Taraxacum officinale* ^123^ and alligator weed *Alternanthera philoxeroides*,^124^ epigenetic marks arising from stress are inherited across multiple generations. Whereas sexual reproduction effectively erases most of the drought‐induced epigenome changes in subsequent generations of *Arabidopsis*,^125^ parental exposure to pathogen attack led to enhanced pathogen resistance in the immediate next generation, likely through transgenerational inheritance of specific DNA and histone epi‐marks.^126^ Recent development of CRISPR/Cas9‐based epigenome‐editing tools therefore affords a new avenue to alter crop metabolic traits or stress resistance without changing the DNA sequence.^127^

Another epigenetic mechanism relevant to climate change is RNA secondary structure dynamics.^128^, ^129^ In particular, we may expect RNA secondary structures to be influenced by environmental factors such as temperature, leading to differential functional outputs.^130^ In fact, it has been shown that mRNA structure is involved in the cold shock response in bacteria.^131^ In plants, early study of the maize (*Zea mays*) transcriptional activator *Lc* uncovered a role of mRNA secondary structure in regulating anthocyanin biosynthesis through translational repression.^132^ A more recent in vivo genome‐wide survey of RNA secondary structure in *Arabidopsis* further revealed that, while genes involved in fundamental cellular maintenance display more confined and predictable mRNA secondary structures, stress‐induced genes contain more plastic mRNA secondary structures likely associated with regulatory functions under different environmental conditions.^133^, ^134^ Understanding the structure‐function relationship of the RNA secondary structures in regulating gene function in response to environmental changes can provide additional tools for precision engineering of certain traits tailored to specific growth conditions.

## 6 OUTLOOK

Global and regional climate changes over the past 470 million years have profoundly shaped the evolution of the Earth's flora. Although all extant plant species have adapted to historical climate changes, including those catastrophic periods that led to mass extinction events,^135^ rapid anthropogenic climate change in the past two centuries may present another unprecedented challenge. Certainly, new cycles of natural selection will continue to select the fittest plants to survive the new environments, but humans have now become a driving force in shaping future plant evolution, both through our desire to retain valuable plant traits, and our increasing capability to modify plants.

Many novel technologies can be readily deployed to help expand our understanding of how diverse plants respond to complex and rapid climate changes, including single‐cell RNA sequencing (scRNA‐seq),^136^ ribosome profiling,^137^ and single‐cell proteomics and metabolomics (Figure 2E.^138^, ^139^ Moreover, the ability to quantitatively assess metabolic states of various cellular compartments in defined tissues^140^, ^141^ will further advance our knowledge on plant organellar responses to environmental changes.^142^ Armed with the growing toolsets of genome editing,^143^ synthetic biology,^144^ and ethically aware regulation of technology,^145^ we can, and shall responsibly manipulate plants to provide commercial, medicinal, or other specialized values with built‐in resilience in the face of climate change. New knowledge and engineered plants arising from these studies may be further applied to mitigate the increasing demand for plant biomass resulting from the current rapid expansion of the human population.^146^ Considering that a few crop species have arisen as the most successful terrestrial plants due to human selection and facilitation, it is foreseeable that new generations of engineered plants with various desirable traits—either bred, edited, or even created from scratch—will arise to accompany future human life on Earth. Existing, preliminary, planned and future efforts to understand and augment our repertoire of plants and their mechanisms of resilience will continue to serve humanity into future generations.

## CONFLICT OF INTEREST

J.K.W. is a co‐founder, a member of the Scientific Advisory Board and a shareholder of DoubleRainbow Biosciences, which develops biotechnologies related to natural products.

## AUTHOR CONTRIBUTIONS

**Sophia Xu:** Conceptualization; writing‐original draft; writing‐review and editing. **Jing‐Ke Weng:** Conceptualization; writing‐original draft; writing‐review and editing.

### PEER REVIEW

The peer review history for this article is available at https://publons.com/publon/10.1002/ggn2.10022.

## Supporting information

Transparent‐Peer‐Review‐Record
