# Supplementary material for: Climate change shapes the future evolution of plant metabolism
Source: Adv Genet (Hoboken). 2020 Aug 10;1(1):e10022. doi: 10.1002/ggn2.10022 (PMC9744464; doi:10.1002/ggn2.10022)
Supplement: Supplementary file 1 — Transparent‐Peer‐Review‐Record [file GGN2-1-e10022-s001.pdf]

Advanced Genetics

Climate change shapes the future evolution of plant metabolism

Sophia Y. Xu and Jing-Ke Weng

Review timeline

Date submitted: 11/20/2019
1st Editorial Decision: Minor Revision - 01/14/2020
Revision received: 01/28/2020
2nd Editorial decision: Minor revision - 02/06/2020
Revision Received: 02/13/2020
Date accepted: 03/02/2020

Editor: Alison Liu

Initial Editorial Evaluation 08/12/2020

---

(1) Can you please introduce a sentence about the solutions that genetics and genomics bring to the issue into your abstract? We need to have a solution to the problem in the abstract, and we cannot wander around other disciplines. As you concluded, either that we need genetic markers to breed plants faster or that natural breeding is not fast enough to introduce the traits required and therefore parallel editing is needed - synthetic biology and genome engineering are good approaches!

(2) Please mainly focus on the secondary metabolite synthesis.

(3) How robust of these metabolite synthetic pathways are in response to environmental changes? How are the levels of synthetic enzymes regulated normally or in response to abiotic stress such as climate conditions? Through gene expression and epigenetic alterations?

(4) How are enzymatic activities altered CHEMICALLY in response to abiotic stress? Are there chemical characteristics of synthetic enzymes vulnerable to elevated temperature and other stressors?

(5) How are the alterations of signal transduction pathways in response to environmental changes transmitted to these metabolic pathways?

(6) Since secondary metabolites are synthesized for plant defense, are these mechanisms already installed in plants? For example, plants routinely respond to daily changes of temperature and seasonal changes of temperature. Since elevated CO_2 was relatively stable in the past million years, do plants have a mechanism to deal with increasing CO_2 and high-levels of carbohydrates? How do sugar levels affect secondary metabolites?

1st Reviews, Editor Decision, and Authors' Response

1st Editorial Decision 01/14/2020

Manuscript ID GGN-2019-0009 entitled "Climate change shapes the future evolution of plant metabolism" which you submitted to Genetics & Genomics Next, has been reviewed very favorably and minor revisions have been requested. I invite you to respond to the comments appended below and revise your manuscript.

Editor Comments to Author: Please address all reviewers' comments and add one additional section in the manuscript according to the reviewer 1, "One related topic that I found to be missing here to be clearly stated is the rapid growth of the world's human population and challenges arising from it. The need to drastically increase global food production will require to find ways to fast adapt crops to environmental stresses and to further increase crop yields, which intersects with the challenges posed by climate change. I wonder if the authors might find a place in the manuscript to emphasize this issue with a sentence or two". This content was in fact mentioned in your abstract, but not included in the manuscript, "We explore potential avenues of future scientific investigations, powered by cutting-edge methods such as synthetic biology and genome engineering, to better understand and mitigate the consequences of rapid climate change on plant fitness and plant usage by humans".

Reviewer(s)' Comments to Author:

Reviewer: 1

Comments to the Author

This review discusses possible impacts of climate change on plants and how plant science can mitigate negative effects on crop productivity.

The authors start by describing the general significance of plants and plant products for human life. They point out several plant secondary metabolites with medical uses. A short overview of earth's climate history within the 500 million years, where evolution of land plants took place, makes clear that Earth's flora had to adapt many times to drastic environmental changes. Yet, recent human activity caused a very fast rise in CO_2. The increase in CO_2 impacts the climate, which poses a challenge to plant growth. Temperature, humidity, ground ozone and UV radiation are identified as major factors that change rapidly and have an effect on plant growth. The balance between growth and defense, which humans have changed in crops to make them more useful, is majorly disturbed. The change in climate has a noticeable Influence on quality and flavor of crop plants adapted to specific environments.

While there is a positive impact of increased CO_2 levels on plant growth, negative impacts of climate change might outweigh these in many cases. To be able to better address these issues, it is of increasing interest to better understand the growth-defense antagonism in crop plants, which is being disturbed. Also, to be able to adapt crops to climate change, it might be important to further study and harness existing plant diversity in order to identify mechanisms for adaptation that could be transferred to crop plants. The authors point out that some of the less understood fields of research relevant to adaptations of plants to changing environment are epigenetic mechanisms and RNA secondary structure and their impact on gene expression. The authors conclude in highlighting recent technologies in biochemical and molecular analytics, genome editing and synthetic biology as tools that can be used to better understand the biology of the plants that humanity relies on in order to address the challenges that climate change poses.

Altogether the manuscript is a well written overview of the current understanding of the impacts of climate change on plants humans rely on for food and many other uses. It is a good reference leading the reader into current topics in plant science that might be important to answer the challenges.

One related topic that I found to be missing here to be clearly stated is the rapid growth of the world's human population and challenges arising from it. The need to drastically increase global food production will require to find ways to fast adapt crops to environmental stresses and to further increase crop yields, which intersects with the challenges posed by climate change. I wonder if the authors might find a place in the manuscript to emphasize this issue with a sentence or two.

Reviewer: 2

Comments to the Author

This perspective review provides an overview of the impact of climate change on crop physiology, metabolism and

productivity. The authors also briefly discuss the prospects that synthetic biology has to offer to alleviate some of the corresponding negative effects.
Overall, the review is well written. It is intended for a layman -though scientifically literate- audience rather than to experts on the topic. The manuscript does not address any concepts or opinions that one hasn't already read or heard about, but the references are appropriate and up to date. In short, this is a decent summary of the mainstream opinions on this subject.

A few points to consider though:

p6: It seems odd to start the section describing climate change as preceding mass extinction of plant species and endangering human existence by 'The good news is that [...] plants have certainly tolerated and overcome more extreme challenges than present day temperatures and CO2 levels'. I suggest to remove 'The good news is that'.

p7: I would remove 'Luckily' (there is nothing 'lucky' or to be proud of in having humans contributing to the current climate crisis; science and technological advances have a major responsibility in such a crisis).
Also, there is not one human society, but some human societies.

p7: Not all phenylpropanoids are specialized metabolites. For instance t-cinnamate and p-coumarate are vital to plant life (e.g. null mutants of cinnamate-4-hydroxylase are lethal); they are primary metabolites.

p15: The last sentence of the paper is odd too ['Although this seems like a sound plan to cope with the changing climate and to keep up with the exponential growth of the human society, we must ask ourselves, is this where we really want to go? The choice in our hands [sic].'].
Since climate change is already happening, what choices does humanity has other than breeding and engineering plants with corresponding desirable traits?

Panels a, b, c, d of Figure 1 are not called in the text in the order they are presented (e.g. 1C is called before a and b). Although this eventually remains an editorial choice, I suggest to re-order the panels in Figure 1, so those match the text.

Panel 1d is non-informative (and the drawings not publication grade); I would delete it

---

1st Author Response to Reviewers and Editor 01/28/2020

Reviewer: 1
Comments to the Author
This review discusses possible impacts of climate change on plants and how plant science can mitigate negative effects on crop productivity.
The authors start by describing the general significance of plants and plant products for human life. They point out several plant secondary metabolites with medical uses. A short overview of earth's climate history within the 500 million years, where evolution of land plants took place, makes clear that Earth's flora had to adapt many times to drastic environmental changes. Yet, recent human activity caused a very fast rise in CO2. The increase in CO2 impacts the climate, which poses a challenge to plant growth. Temperature, humidity, ground ozone and UV radiation are identified as major factors that change rapidly and have an effect on plant growth. The balance between growth and defense, which humans have changed in crops to make them more useful, is majorly

disturbed. The change in climate has a noticeable Influence on quality and flavor of crop plants adapted to specific environments.
While there is a positive impact of increased CO2 levels on plant growth, negative impacts of climate change might outweigh these in many cases. To be able to better address these issues, it is of increasing interest to better understand the growth-defense antagonism in crop plants, which is being disturbed. Also, to be able to adapt crops to climate change, it might be important to further study and harness existing plant diversity in order to identify mechanisms for adaptation that could be transferred to crop plants. The authors point out that some of the less understood fields of research relevant to adaptations of plants to changing environment are epigenetic mechanisms and RNA secondary structure and their impact on gene expression. The authors conclude in highlighting recent technologies in biochemical and molecular analytics, genome editing and synthetic biology as tools that can be used to better understand the biology of the plants that humanity relies on in order to address the challenges that climate change poses.
Altogether the manuscript is a well written overview of the current understanding of the impacts of climate change on plants humans rely on for food and many other uses. It is a good reference leading the reader into current topics in plant science that might be important to answer the challenges.
One related topic that I found to be missing here to be clearly stated is the rapid growth of the world's human population and challenges arising from it. The need to drastically increase global food production will require to find ways to fast adapt crops to environmental stresses and to further increase crop yields, which intersects with the challenges posed by climate change. I wonder if the authors might find a place in the manuscript to emphasize this issue with a sentence or two.

We thank Reviewer 1 for her/his thoughtful content suggestions. We found it a relevant consideration for future work and have added a sentence in that section.

Reviewer: 2
Comments to the Author
This perspective review provides an overview of the impact of climate change on crop physiology, metabolism and productivity. The authors also briefly discuss the prospects that synthetic biology has to offer to alleviate some of the corresponding negative effects.
Overall, the review is well written. It is intended for a layman -though scientifically literate- audience rather than to experts on the topic. The manuscript does not address any concepts or opinions that one hasn't already read or heard about, but the references are appropriate and up to date. In short, this is a decent summary of the mainstream opinions on this subject.

A few points to consider though:

p6: It seems odd to start the section describing climate change as preceding mass extinction of plant species and endangering human existence by 'The good news is that [...] plants have certainly tolerated and overcome more extreme challenges than present day temperatures and CO2 levels'. I suggest to remove 'The good news is that'.

We have revised accordingly.

p7: I would remove 'Luckily' (there is nothing 'lucky' or to be proud of in having humans contributing to the current climate crisis; science and technological advances have a major responsibility in such a crisis).
Also, there is not one human society, but some human societies.

We have revised accordingly.

p7: Not all phenylpropanoids are specialized metabolites. For instance t-cinnamate and p-coumarate are vital to plant life (e.g. null mutants of cinnamate-4-hydroxylase are lethal); they are primary metabolites.

Regarding the light-induced phenylpropanoids (p7), we have adjusted the wording to refer more accurately reflect the specialized phenylpropanoids discussed in the two references.

p15: The last sentence of the paper is odd too ['Although this seems like a sound plan to cope with the changing climate and to keep up with the exponential growth of the human society, we must ask ourselves, is this where we really want to go? The choice in our hands [sic].'].
Since climate change is already happening, what choices does humanity has other than breeding and engineering plants with corresponding desirable traits?

Thanks for the suggestion. We have revised the last sentence to "Existing, preliminary, planned and future efforts to understand and augment our repertoire of plants and their mechanisms of resilience will continue to serve humanity into future generations."

Panels a, b, c, d of Figure 1 are not called in the text in the order they are presented (e.g. 1C is called before a and b). Although this eventually remains an editorial choice, I suggest to re-order the panels in Figure 1, so those match the text.

Panel 1d is non-informative (and the drawings not publication grade); I would delete it.

Regarding Figure 1, we see the point that Panel D did not add anything to the figure, and have implemented your suggestions to remove that panel and rearranged the order of Panels A, B and C.

2nd Editorial Decision 02/06/2020

We recommend adding a table with the secondary metabolites and their related regulatory pathways in the tradeoff between the production and defense in response to various climate change conditions. You discussed about them in the text, and a table can help strengthen your points. We suggest that you organize four main classes of specialized metabolite compounds, terpenoids, phenolic compounds, alkaloids, and sulphur-containing compounds, using internationally recognized chemical identifiers (INCHI or SMILE) or ontological terms. Ontology supports for chemical entities, molecular functions and interactions and plant environmental conditions:
https://www.ebi.ac.uk/ols/index
http://www.ontobee.org
https://bioportal.bioontology.org
https://pubchem.ncbi.nlm.nih.gov
https://www.ebi.ac.uk/chebi/

Also, increasing levels of atmospheric CO_2 can have another detrimental effect on plants by altering their flowering time. This has been demonstrated in many studies. Here are some papers: 60 studies reviewed by Springer and Ward, 2007, https://nph.onlinelibrary.wiley.com/doi/full/10.1111/j.1469-8137.2007.02196.x; Kazan and Lyons, 2016, doi:10.1093/jxb/erv441; Cui et al, 2014, doi: 10.1111/tpj.12712; Becklin et al, 2017, doi:

10.1111/nph.14336; Walker and Ward, 2018; doi: 10.1007/s00442-018-4197-0. Other studies have shown that elevated CO2 might affect plant flowering time through regulating miRNAs (May et al, 2013, https://doi.org/10.1038/ncomms3145; Saminathan et al, 2019, doi: 10.1007/s10142-018-0635-7; Tripathi et al. 2019, doi: 10.1038/s41598-018-37465-y). As you know, both plant flowering time and metabolism are extremely responsive to environmental stress, these studies suggest that photosynthetic sugar and sugar metabolites can regulate miRNAs-regulated flowering regulatory pathways (Yu et al. 2013, https://doi.org/10.7554/eLife.00269.001; Yang et al. 2013, https://doi.org/10.7554/eLife.00260.001; Wahl et al. 2013, https://doi.org/10.1126/science.1230406) and other juvenile-to-adult transition pathways (Matsoukas et al. 2013, https://doi.org/10.1111/pce.12088). However, we leave this for you to decide, in case that you want to comment on this topic, how to cite the references.

Finally, as you must be aware, there are controversies regarding genetic engineering of crops and other lives, including humans, so proper regulations are needed for the safety measure. We recommend including this message, perhaps in the last paragraph - you may like to cite this reference on the ethical implementation of safe genome editing in crop plants (Huang et al, 2016, https://doi.org/10.1038/ng.3484).

2nd Author Response to Editor 02/13/2020

Thank you for revising your manuscript. It reads great! Before sending it to our production team, we recommend some minor edits in the text (see the track changes in the attached manuscript). Please also use numbers for your cited references in the text to fit our journal style, following our guide for authors.

We have adjusted our references accordingly.

We recommend adding a table with the secondary metabolites and their related regulatory pathways in the tradeoff between the production and defense in response to various climate change conditions. You discussed about them in the text, and a table can help strengthen your points. We suggest that you organize four main classes of specialized metabolite compounds, terpenoids, phenolic compounds, alkaloids, and sulphur-containing compounds, using internationally recognized chemical identifiers (INCHI or SMILE) or ontological terms. Ontology supports for chemical entities, molecular functions and interactions and plant environmental conditions: https://www.ebi.ac.uk/ols/index, http://www.ontobee.org, https://bioportal.bioontology.org https://pubchem.ncbi.nlm.nih.gov, https://www.ebi.ac.uk/chebi/

While we see that a visual reference in the form of a table might be a useful tool for readers to better understand the material, we believe that including such a table in the present manuscript will not add value, and in fact, may mislead readers. To elaborate: a closer inspection of the growth-defense balance section reveals that we mentioned only three plant hormones, which seems insufficient to build a table around. If, instead, the request was for us to include all metabolites from the entire paper to be included in the table, we believe that such a compilation would be better left for a more thorough review, such as in Annual Reviews. This is because, in our manuscript, we discuss only scattered studies, and presenting this in a table may give the misimpression that the contents are a holistic review of the field, which is not true.

Also, increasing levels of atmospheric CO2 can have another detrimental effect on plants by altering their flowering time. This has been demonstrated in many studies. Here are some papers: 60 studies reviewed by Springer and Ward, 2007, https://nph.onlinelibrary.wiley.com/doi/full/10.1111/j.1469-8137.2007.02196.x; Kazan and Lyons, 2016, doi:10.1093/jxb/erv441; Cui et al, 2014, doi: 10.1111/tpj.12712; Becklin et al, 2017, doi: 10.1111/nph.14336; Walker and Ward, 2018; doi: 10.1007/s00442-018-4197-0. Other studies have shown that elevated CO2 might affect plant flowering time through regulating miRNAs (May et al, 2013, https://doi.org/10.1038/ncomms3145; Saminathan et al, 2019, doi: 10.1007/s10142-018-0635-7; Tripathi et al.

2019, doi: 10.1038/s41598-018-37465-y). As you know, both plant flowering time and metabolism are extremely responsive to environmental stress, these studies suggest that photosynthetic sugar and sugar metabolites can regulate miRNAs-regulated flowering regulatory pathways (Yu et al. 2013, https://doi.org/10.7554/eLife.00269.001; Yang et al. 2013, https://doi.org/10.7554/eLife.00260.001; Wahl et al. 2013, https://doi.org/10.1126/science.1230406) and other juvenile-to-adult transition pathways (Matsoukas et al. 2013, https://doi.org/10.1111/pce.12088). However, we leave this for you to decide, in case that you want to comment on this topic, how to cite the references.

This is an interesting and important topic, but we believe it (and its corresponding primary metabolites) fall somewhat outside the scope of our paper. Nevertheless, we have included a sentence about the effect of climate change on flowering time in the introduction, and have included a subset of the references you suggested for further reading.

Finally, as you must be aware, there are controversies regarding genetic engineering of crops and other lives, including humans, so proper regulations are needed for the safety measure. We recommend including this message, perhaps in the last paragraph - you may like to cite this reference on the ethical implementation of safe genome editing in crop plants (Huang et al, 2016, https://doi.org/10.1038/ng.3484).

We agree that the ethical and responsible application of genome editing technologies is an important message to promote. However, we have moved this message and its corresponding reference out of the very last sentence of the paper, into the middle of the same paragraph as a stylistic choice.
